# Cardiovascular Risk in Patients with Chronic Hepatitis C Treated with Direct Acting Antivirals

**DOI:** 10.3390/jcm11195781

**Published:** 2022-09-29

**Authors:** Mohammad Said Ramadan, Filomena Boccia, Simona Maria Moretto, Fabrizio De Gregorio, Massimo Gagliardi, Domenico Iossa, Emanuele Durante-Mangoni, Rosa Zampino

**Affiliations:** 1 Department of Precision Medicine, University of Campania ‘L. Vanvitelli’ Napoli, 80138 Naples, Italy; 2 Department of Advanced Medical and Surgical Sciences, University of Campania ‘L. Vanvitelli’, 81031 Naples, Italy; 3 Unit of Infectious and Transplant Medicine, AORN Ospedali dei Colli-Monaldi Hospital, Piazzale E. Ruggieri, 80131 Naples, Italy

**Keywords:** liver fibrosis, cardiovascular disease, direct acting antivirals, atherosclerotic cardiovascular disease score, DAA

## Abstract

Background: Chronic hepatitis C (CHC) is associated with hepatic and extrahepatic complications, including cardiovascular disease (CVD). The effects of sustained virological response (SVR) and liver fibrosis on CVD risk are not well established. Aims: We aim to assess the dynamics of Fibrosis-4 (FIB-4) and Atherosclerotic Cardiovascular Disease 2013 (ASCVD) scores up to three years after direct acting antivirals (DAA) treatment and explore the time-dependent association between the two scores. Methods: We included consecutive CHC patients treated with DAA and followed up with them for three years. Outcomes were changes from baseline (before DAA) in ASCVD and FIB-4 scores, measured at the end of treatment, 12-, 24-, and 36-months follow-up. Results: In total, 91 patients with CHC were finally included (median age: 66 years (IQR = 58–72 years); 43% females). Median follow-up was 2 years (1–3 years) and all patients reached SVR. The ASCVD score did not significantly change from baseline (Mean = 17.2%, 95% CI 14.1, 20.3), but the FIB-4 score significantly decreased at any time-point by an average of 0.8 (95% CI 0.78, 0.82, *p* < 0.001). Elevated FIB-4 scores at one (β = 1.16, *p* < 0.001) and three years (β = 2.52, *p* < 0.001) were associated with an increased ASCVD score. Clinically, two participants- with non-decreasing FIB-4 scores after treatment- had acute coronary syndrome at the end of treatment and one year follow-up, respectively. Conclusions: In our study, we found that FIB-4 and ASCVD scores exhibited a positive correlation irrespective of time-point after treatment. Larger studies are essential to further investigate the utility of FIB-4 scores in cardiovascular risk assessment.

## 1. Introduction

Chronic hepatitis C (CHC) remains a major cause of liver-associated morbidity and mortality globally [1] with more than 50 million affected individuals worldwide [2]. CHC usually follows acute hepatitis C virus (HCV) infection in most patients [3] and is increasingly considered a systemic disease that goes beyond its hepatic manifestations [4,5,6,7]. 

Although liver complications, such as liver failure, fibrosis, and hepatocellular carcinoma account for most of the morbidity and mortality in CHC, serious extrahepatic sequelae, including those related to the cardiovascular system, are increasingly investigated [8,9]. These include atherosclerotic cardiovascular disease, dyslipidemia, and glucose metabolic derangement/diabetes mellitus [4,5,6]. A meta-analysis of 22 studies found that patients with CHC had 1.6-, 2.2-, and 1.3-times increased risk of cardiovascular disease (CVD) related mortality, carotid plaques, and cerebrocardiovascular events, respectively, compared to uninfected individuals [10]. In addition to mortality, HCV-related CVD was shown to be associated with 1.5 million disability-adjusted life-years (DALYs) in a recent meta-analysis of 341,739 people with HCV [11].

Mechanisms underlying the pathogenesis of CHC-associated CVD are not completely understood [12]. Suggested mediators include promoted systemic inflammation and thrombosis and altered glucose and lipid metabolism. These increase the risk for metabolic diseases including both steatosis and diabetes, and thus CVD [4,13,14]. CHC was shown to be associated with higher levels of pro-inflammatory markers such as C-reactive protein and fibrinogen [15], thereby promoting systemic inflammation, a central process in the development of coronary heart disease and atherosclerosis [6,16]. Moreover, HCV was isolated from carotid plaques, suggesting it could also directly cause local infection, promoting atherosclerosis and CVD [17]. 

Direct acting antivirals (DAAs), the current treatment of choice for CHC, have minimal side effects, short treatment duration, and provide higher than 95% efficacy in establishing sustained virological response (SVR) [18,19,20]. DAAs were shown to reduce CHC hepatic complications, overall mortality, and need for liver transplantation [6,12,21]. However, the effect on CHC-associated CVD is not as clear [22]. Several studies showed reduced CVD, including acute coronary syndrome, atherosclerosis, and type 2 diabetes, with DAA treatment and SVR, as compared to no treatment or no SVR [23,24,25]. However, other studies failed to reach a similar conclusion, where participants were found to have worsening lipid profiles (such as increased total cholesterol and LDL cholesterol levels) [26,27,28,29,30,31], worsening or no improvement in insulin resistance [27,32] and liver steatosis [28,29], and increased CVD events after treatment [31] (Table S1).

In the present study, we aimed to investigate cardiovascular risk after DAA treatment according to the parallel dynamics of liver fibrosis.

## 2. Materials and Methods

### 2.1. Patients and Study Design

**Figure 1:**
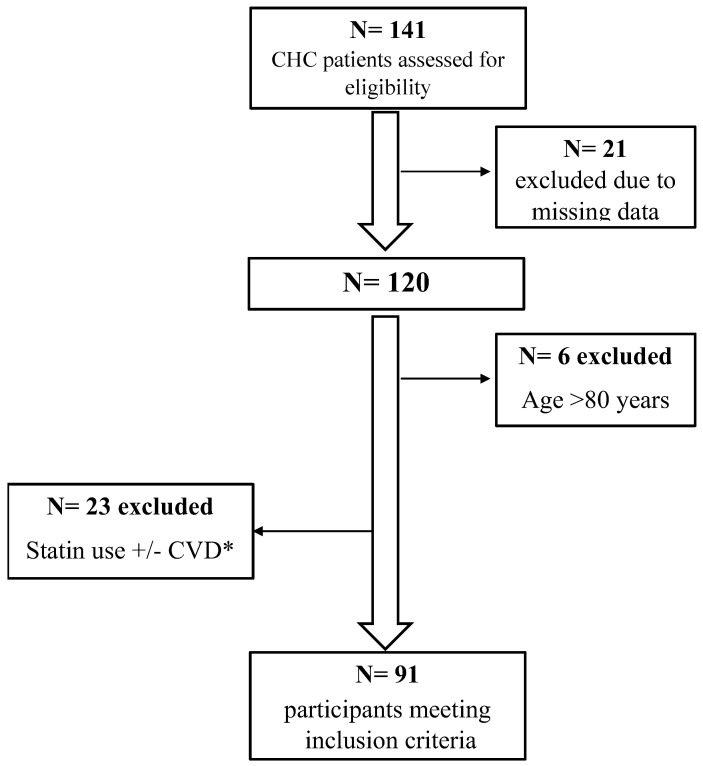
Flow-chart of the study. ACS, acute coronary syndrome; CHC, chronic hepatitis C; CVD, cardiovascular disease; CVA, cerebrovascular accident; * groups not mutually exclusive: N = 7: previous ACS, N = 2: previous CVA, N = 10: cardiac surgery, N = 2: heart failure, N = 7 concurrent statin use.

In this study, we retrospectively included consecutive adult patients with CHC treated with DAA at the Unit of Infectious and Transplant Medicine, University of Campania “Luigi Vanvitelli”, Monaldi Hospital in Naples, Italy, from 2015 to 2020, and data was retrieved up to three years after the initial encounter. Patients with decompensated cirrhosis, missing data, aged less than 39 years or greater than 79 years, established prior cardiovascular events, or who were receiving lipid lowering therapy before DAA treatment were excluded (Figure 1). DAA treatment was administered in accordance with the European Association for the Study of the Liver [33] and Italian Medicines Agency (AIFA) Guidelines [34] and their subsequent updates over time [35,36].

The study was approved by the Ethics Committee of University of Campania “Luigi Vanvitelli (decision n. 662/2017). Personal and clinical data were managed in agreement with the Declaration of Helsinki and the General Data Protection Regulation (679/2016). All patients provided informed consent to participate in the study. We present this study in accordance with the Strengthening the Reporting of Observational studies in Epidemiology (STROBE) guidelines (Table S2) [37].

### 2.2. Definition and Ascertainment of Outcomes

As part of standard management, patients underwent a series of hemato-chemical and clinical evaluations before, by the end of, and after DAA treatment. Laboratory tests were performed by standard hospital procedures; similar and validated tools were used to measure biometric data, including blood pressure and weight, when required. Response to antiviral treatment was defined as SVR (undetectable HCV RNA) at 12 (SVR12) or 24 (SVR24) weeks after the end of therapy [38].

We used the Atherosclerotic Cardiovascular Disease 2013 (ASCVD) Risk Calculator [39] to estimate ASCVD risk throughout the follow-up period. This score estimates the 10-year risk of a first-time episode of coronary heart disease (CHD) or stroke among patients 40–79 years of age. Borderline risk was defined as 5% to <7.5%, intermediate risk as 7.5% to <20%, and high risk was defined as ≥20% [40]. 

### 2.3. Data Collection 

Data was collected retrospectively starting from before DAA treatment and up to 3 years afterwards, with one evaluation added per year, when available. Collected clinical variables included blood pressure, weight, height, body mass index (BMI), and personal and family history (with particular attention to alcohol intake and CVD). Lab data included HCV viral load and genotype, liver function tests (liver enzymes, bilirubin, albumin), complete blood count, glucose metabolism (HbA1c, fasting blood glucose (FBG)), renal function tests (creatinine, BUN), and lipid panel (cholesterol, LDL, HDL, triglycerides). Liver enzyme levels were analyzed according to the upper limit of the normal range (ULN). We calculated the Fibrosis-4 (FIB-4) noninvasive score [41] to estimate liver fibrosis, including cirrhosis. FIB-4 index was calculated according to this formula: (aspartate aminotransferase (AST) [IU/L] × age [years])/(platelet count [10^9^/L] × alanine aminotransferase (ALT) [IU/L]^1/2^). Evaluation of liver fibrosis, done by transient elastography at baseline (TE, FibroScan^®^, Paris, France), was categorized into: F0–F1 ([2–7] kPa), F2 ([8–9[ kPa), F3 ([9–14[), and F4 (≥14 kPa) [42]. Evaluation of liver steatosis was determined by observation of bright liver echo pattern on ultrasound.

### 2.4. Sample Size Calculation

Details of sample size calculation are presented in Text S1. First, we calculated the sample size required to detect a mean difference in the ASCVD score of 5 (effect size = 0.42, medium effect) using a paired *t* test with 80% power at significance of alpha = 0.05. We then assumed 15% loss to follow-up rate and adjusted the calculation for correlated measures (assumed to equal 0.5). The final required sample size per time-point was 66 patients.

### 2.5. Statistical Analysis 

Continuous variables were expressed as medians and interquartile range (IQR), whereas categorical variables were presented as values and percentages. Continuous variables were compared using the Kruskal–Wallis or Mann–Whitney U tests, whereas categorical variables were compared using Chi-squared or Fisher’s exact test, as applicable. No imputation for missing data was done, to avoid introducing a bias to the results. A two-sided probability *p*-value < 0.05 was considered statistically significant. Subgroup analysis was done for patients with CV events.

Linear regression models based on generalized estimating equations (GEE) for repeated measurements were used to compare ASCVD values before and after DAA treatment in all participants. Participant had 5 outcome measures (before, directly after, and at 1, 2, 3 years after DAA treatment) and the primary predictors were follow-up year, FIB-4 score and interactions between these two predictors. The model was adjusted for renal function [43] and BMI [44]; variables excluded from the model included those used to calculate the ASCVD score (such as age, gender, total cholesterol…). These models allowed us to determine if the ASCVD score, at each follow-up, significantly changed from baseline. In addition, to explore the effect of the FIB-4 score on ASCVD across the different time points, we included a time-varying variable for FIB-4 in the GEE. 

Summary tables were created using gtsummary [45] and dplyr packages [46]. Plots, sample size calculation, and linear models were done using ggplot2 [47], pwr [48], and geepack [49] packages, respectively. All analyses were done in R version 4.1.2 (R & Company, Vienna, Austria) [50].

## 3. Results

### 3.1. Study Subjects’ Characteristics

Demographic, clinical characteristics, and hemato-chemical data before and after DAA treatment are presented in Table 1. A total of 91 patients (Figure 1) with CHC were finally included in the analysis (median age: 66 years (58–72 years); 43% females) and most had HCV genotype 1b (62%). Median follow-up was 2 years (IQR= 1–3 years), and all patients reached SVR. Demographic, clinical characteristics, and hemato-chemical data before and after DAA treatment are presented in Table 1. Types of DAA therapy used are presented in Table S3.

The major cardiometabolic parameters remained stable over the observation period. Number of participants reaching one, two, and three years of follow up after DAA treatment was 88, 81, and 60, respectively. The reason for this is due to the difference in the time of treatment initiation between patients. Missing values for variables are presented in Table S4.

### 3.2. Changes in Liver Fibrosis after DAA Treatment

Detailed liver function tests and FIB-4 score results are shown in Table 2. More than half of the included individuals had FibroScan levels >8 kPa, indicating a liver fibrosis stage of F2 (significant fibrosis) or higher, at baseline. This was also suggested by an elevated FIB-4 score at baseline (2.27, (1.63–4.11)) (Table 2), the latter correlating moderately with baseline FibroScan levels (r = 0.42, *p* < 0.001).

Before DAA treatment, 24%, 41%, and 33% of the patients had elevated ALT, AST, and GGT levels, respectively. However, these normalized in almost all participants, starting from directly after treatment (*p* < 0.001) and until the end of the study. 

### 3.3. Dynamics of ASCVD and FIB-4 Scores over Time

**Figure 2:**
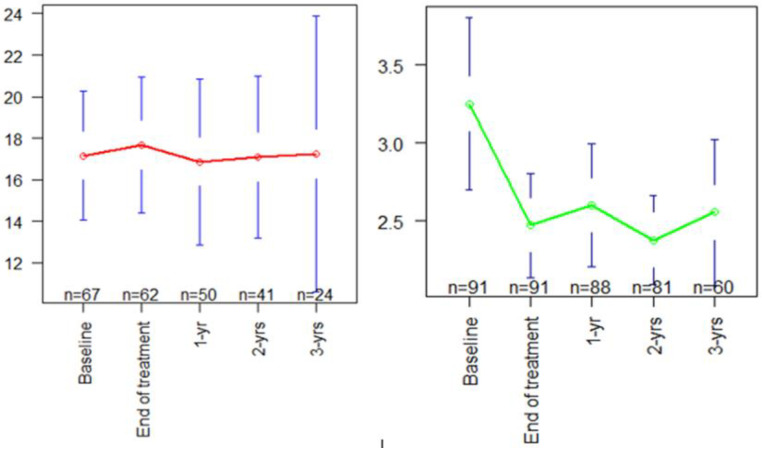
Dynamics of ASCVD and FIB-4 scores with time at baseline and follow-up (mean [95%CI]). After DAA treatment, mean ASCVD scores (in red) did not show significant change from baseline, whereas mean FIB-4 scores (in green) abruptly decreased starting at the end of treatment. ASCVD, Atherosclerotic Cardiovascular Disease 2013; FIB-4, fibrosis-4 index.

The ASCVD score was available for 67 (74%), 62 (68%), 50 (57%), 41 (51%), and 24 (40%) patients at baseline, end of treatment, and 1, 2, and 3-years after treatment (Figure 2). Reasons for the missing ASCVD score were mostly related to missing cholesterol levels (Table S4). Mean ASCVD scores did not significantly change from baseline (Mean = 17.2%, 95% CI 14.1, 20.3) to follow-up time-points (Figure 2 and Table 3). This contrasted with FIB-4 scores, whose baseline means significantly decreased by 0.83 (95% CI 0.18, 1.48, *p* < 0.001), 0.69 (95% CI 0.01,1.37, *p* < 0.001), 0.91 (95% CI 0.29,1.54, *p* < 0.001) and 0.76 (95% CI 0.03,1.48, *p* < 0.001), at the end of treatment, one-, two-, and three-year follow-up, respectively. More specifically, the FIB-4 score decreased in 49 (54%), remained stable in 10 (11%), and increased non-significantly in 32 (35%) patients 1-year after treatment, compared to before treatment. ASCVD scores among participants with non-decreasing FIB-4 (*n* = 42, 46%) are presented in Table S5.

Two participants had a myocardial infarction, one of whom at the end of treatment and the other at the one-year follow-up (baseline ASCVD and FIB-4 scores: 17.9% and 6.4%, 1.4 and 1.9, respectively, Table S6). No other cardiovascular events—including cerebrovascular and acute coronary syndrome events—were recorded throughout the time period.

### 3.4. Association between ASCVD and FIB-4 Scores

After adjusting for BMI, FIB-4 score, and creatinine levels, the ASCVD score did not show any significant change from baseline values to the subsequent time points (Table 4). Participants with creatinine levels >1 mg/dL exhibited elevated ASCVD scores irrespective of the time point (β = 7.3, *p* < 0.01).

Notably, significant interaction was observed between the FIB-4 score and the timeperiod, specifically, increased FIB-4 score at the first and third years after treatment, associated with an increasing ASCVD score (β (year1/baseline) = 1.16, *p* = 0.017; β (year3/baseline) = 2.52, *p* < 0.001).

**Figure 3:**
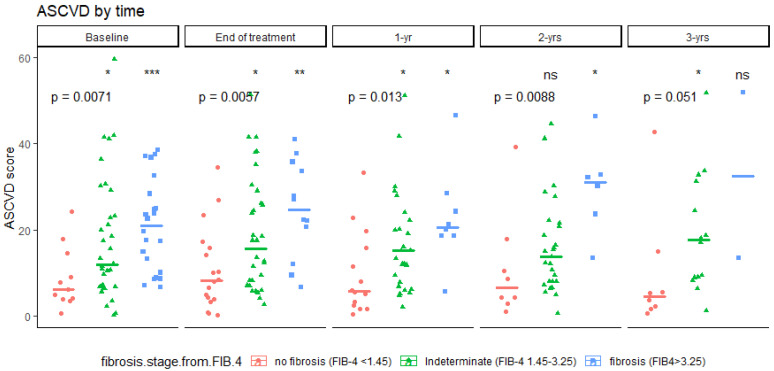
ASCVD score variation according to FIB-4 score groups and time. ASCVD, Atherosclerotic Cardiovascular Disease 2013; FIB-4, fibrosis-4 index. ns: non-significant p-value (>0.05). *: (0.01, 0.05), **: (0.001, 0.01], ***: [0, 0.001], determined by Wilcoxon rank sum test (taking no fibrosis as the reference group). Overall *p*-values calculated using Kruskal–Wallis rank sum test.

At each time point, the ASCVD score was significantly associated with the FIB-4 score (Figure 3). In contrast, no FIB-4 group showed any significant change in ASCVD score over time (Figure S1).

## 4. Discussion

In this study, we longitudinally evaluated the variation of ASCVD and FIB-4 scores, estimating cardiovascular risk and liver fibrosis, respectively, in a cohort of patients with CHC receiving DAA treatment. We also assessed the relationship between time-varying FIB-4 and ASCVD scores.

At baseline, most participants had liver fibrosis (as determined by both TE and FIB-4 score) and moderately elevated ASCVD risk. Over time, we observed a significant decrease in FIB-4 score after DAA (implying decreasing liver fibrosis/inflammation) and no change in ASCVD, despite increased statin use and unchanged cardiovascular risk factors (such as hypertension and T2DM). Importantly, rising FIB-4 values at the first and third years after treatment was significantly associated with an increasing ASCVD score. This trend was also observed in the second year, albeit with no significance (*p* = 0.07), which could be due to the limited sample size. Stable or increasing FIB-4 scores could be explained for other concomitant causes of hepatic injury, or progression of fibrosis [51]. Observed cardiovascular events consisted of two patients developing MI at the end of treatment and at the one-year follow-up, respectively, both with non-decreasing FIB-4 scores. Stable cholesterol levels, despite the increased use of statins, is coherent with previous data showing increased cholesterol levels after HCV eradication [22,26,27]. The effect of statin on fibrosis levels is yet to be clarified; however, some studies showed an association of the former with decreased inflammation and fibrosis [52,53].

Our findings of improved liver outcomes after DAA are consistent with other studies, in which both invasive and non-invasive liver fibrosis scores (such as FIB-4) improved as soon as the end of treatment [54,55,56]. Liver fibrosis is a major determinant of prognosis and mortality in CHC [57,58] and direct visualization by biopsy remains the gold standard for staging. However, biopsy is subject to sampling errors, where different stages could be concluded from different samples [59], is expensive, invasive, and although generally safe, could cause complications in up to 7% of patients [57]. The FIB-4 score represents a non-invasive and highly accurate method of estimating liver fibrosis in CHC patients [41]. In a study by Huang et al., authors compared histological and non-invasive fibrosis (including FIB-4) evaluation both before and after DAA treatment, showing that FIB-4 accurately predicted the fibrosis stage [56]. 

In addition to fibrosis, CHC is associated with elevated risk of coronary artery atherosclerosis [60,61], ischemic stroke [62], and thromboembolic events [63] when compared to uninfected controls. These in turn translate into higher CVD-related mortality and disability [10,11]. In our cohort, clinical events consisted of two episodes of acute coronary syndrome (4.6 events per 1000 patient-years), which is lower than what is reported in other studies for HCV patients (around 16 per 1000 patient years), and for untreated controls (31 per patient years) [64]. This difference could be due to the limited sample size and follow-up time.

CV risk scores as a means to predict CVD in CHC are increasingly investigated, yet have variable results as compared to liver fibrosis scores [43,65,66,67]. For instance, Chew et al. compared ASCVD scores to CV events in CHC patients over a 10-year follow-up and found that it performed well in patients at average risk (defined around 7.5%) but underestimated the risk for those with higher risk [66]. Since most patients in our study had a higher ASCVD score than 7.5%—consistent with studies showing a higher-than-average risk score for CHC patients [67,68]—this could imply an even higher than predicted CV risk in the included patients [66]. Nonetheless, the ASCVD risk score remained constantly elevated over the follow-up period, in agreement with other studies utilizing similar CV risk scores [65]. This finding contrasts with other studies showing declining CV events after DAA treatment [43,69,70], and thus the utility of CVD risk scores in CHC patients deserves further investigations.

Interestingly, we found a significant interaction between time after treatment and FIB-4 score in our model, signifying that an increase in FIB-4 score during the first and third years after treatment is associated with an increase in ASCVD risk. Indeed, one of the two patients with MI at one year post-treatment had an elevated FIB-4 score at all time points after receiving treatment (as compared to baseline), and a parallel elevation in ASCVD score (Table S6). Several studies showed induction of dyslipidemia after DAA, which could explain the increase in ASCVD score in these studies, as the latter includes total cholesterol and HDL-C levels [26,27,28,29,30,31] The association between liver fibrosis and ASCVD could be mediated by several mechanisms including systemic inflammation and altered glucose and lipid metabolism induced by the former, which could present risk factors for developing ASCVD. 

This study has several limitations. First, we used FIB-4 and ASCVD scores for liver fibrosis and CVD estimation, respectively, but the former could misclassify some patients (inherent bias of a non-invasive score) or reflect a reduction in liver inflammation rather than fibrosis, and the latter is not validated in patients with CHC. However, we tried to minimize these biases by including TE evaluation (available only at baseline) and collected clinical cardiovascular events, respectively. Second, both scores have age as a common variable in their respective formulas, which could partially explain the observed correlation. Third, we have a limited sample size, which was below the required to achieve significance in certain analyses, and a medium-term follow-up after treatment; thus, our findings should be reassessed on a longer follow-up time. Fourth, our cohort represents patients from a single geographic area and was treated by different DAA formulations, and so results from our study might not be generalizable to other patients from different geographic regions and/or be affected by the DAA type. Finally, due to the relatively high number of missing values in the ASCVD score and retrospective nature of the study, our results could be biased, and despite the observed association between ASCVD and FIB4 scores, our results do not imply causality between fibrosis and cardiovascular diseases.

## 5. Conclusions

Our results show that there was significant improvement of liver fibrosis after DAA treatment and that increased FIB-4 scores independently correlated with higher ASCVD scores, irrespective of the time point after treatment. Larger studies are essential to further investigate the utility of FIB-4 scores in cardiovascular risk assessment.

## Figures and Tables

**Table 1:** Demographics, Cardiometabolic profile and HCV viremia.

Variable	Baseline ^1^	End of DAA Treatment ^1^	One Year	Two Years	Three Years After DAA ^1^	*p*-Value ^2^
			After DAA ^1^	After DAA ^1^		
**Age** (years)	66 (58, 72)	67 (59, 73)	68 (60, 74)	69 (61, 74)	70 (63, 74)	0.063
**Sex**						0.6
Female	39 (43%)	39 (43%)	38 (43%)	34 (42%)	19 (32%)	
**BMI** (kg/m^2^)	27 (24, 29)	27 (24, 29)	27 (24, 29)	27 (24,29)	27 (25, 30)	>0.9
**Current smoker**	25 (27%)	25 (27%)	25 (28%)	21 (26%)	11 (18%)	0.7
**Alcohol dependence**	8 (9%)	8 (9%)	6 (7%)	4 (5%)	3 (5%)	0.8
**SBP** (mmHg)	130 (120, 140)	130 (120, 140)	130 (120, 140)	135 (124, 140)	130 (120, 140)	0.4
**DBP** (mmHg)	80 (70, 80)	80 (70, 85)	80 (70, 85)	80 (70, 85)	80 (75, 81)	0.6
**T2DM**	23 (19%)	21 (18%)	21 (18%)	23 (22%)	17 (22%)	>0.9
**Antidiabetic treatment**	16 (18%)	15 (16%)	15 (17%)	15 (19%)	12 (20%)	>0.9
**Total Cholesterol**	168 (149, 191)	164 (148, 188)	180 (161, 208)	173 (157, 197)	174 (162, 192)	0.09
**LDL-C level (mg/dL)**	104 (87, 118)	106 (94, 128)	109 (102, 138)	106 (85, 122)	105 (82, 120)	0.4
**HDL-C level (mg/dL)**	52 (42, 63)	50 (42, 59)	51 (45, 61)	51 (41, 60)	54 (45, 63)	0.8
**Hypertension treatment**	57 (63%)	59 (65%)	60 (68%)	58 (72%)	44 (73%)	0.6
**Statin treatment**	0 (0%)	4 (4.4%)	10 (11%)	14 (17%)	16 (27%)	**<0.001**
**HCV genotypes**						-
1a	4 (4.4%)	-	-	-	-	
1b	56 (62%)	-	-	-	-	
2	21 (23%)	-	-	-	-	
3	7 (7.7%)	-	-	-	-	
4	3 (3.3%)	-	-	-	-	
**HCV RNA level** (×10^6^ IU/L)	1.3 (0.5, 4.4)	0 (0, 0)	0 (0, 0)	0 (0, 0)	0 (0, 0)	-
**Steatosis**	32 (35%)	19 (21%)	26 (30%)	27 (33%)	24 (40%)	0.105

^1^ Median (IQR); *n* (%). ^2^ Kruskal–Wallis rank sum test; Pearson’s Chi-squared test; Fisher’s exact test. BMI, body mass index; DAA, Direct acting antivirals; DBP, diastolic blood pressure; HCV, hepatitis C virus; SBP, systolic blood pressure; T2DM: type 2 diabetes mellitus.

**Table 2:** Liver TE (baseline only) and enzymes over follow-up time periods.

Variable	Baseline ^1^	End of DAA Treatment ^1^	One Year after DAA ^1^	Two Years after DAA ^1^	Three Years after DAA ^1^	*p*-Value ^2^
**AST groups**						**<0.001**
≤ULN	54 (59%)	87 (96%)	85 (97%)	81 (100%)	60 (100%)	
>ULN	37 (41%)	4 (4.4%)	3 (3.4%)	0 (0%)	0 (0%)	
**ALT groups**						**<0.001**
≤ULN	69 (76%)	91 (100%)	86 (98%)	81 (100%)	60 (100%)	
>ULN	22 (24%)	0 (0%)	2 (2.3%)	0 (0%)	0 (0%)	
**GGT groups**						**<0.001**
≤ULN	61 (67%)	85 (93%)	82 (93%)	79 (98%)	58 (97%)	
>ULN	30 (33%)	6 (6.6%)	6 (6.8%)	2 (2.5%)	2 (3.3%)	
**Fibrosis scores by transient elastography**						NA
F0–F1	30 (41%)	-	-	-	-	
F2	9 (12%)	-	-	-	-	
F3	15 (21%)	-	-	-	-	
F4	19 (26%)	-	-	-	-	

^1^ Median (IQR); *n* (%). ^2^ Kruskal–Wallis rank sum test; Pearson’s Chi-squared test; Fisher’s exact test. Wilcoxon rank sum test. ALT, alanine aminotransferase; AST, aspartate aminotransferase; GGT, gamma-glutamyl transferase; ULN, upper limit of normal, TE, transient elastography.

**Table 3:** Changes from baseline in ASCVD and FIB-4 scores.

Variable	Time Point	Mean (SD)	Mean Difference from Baseline (95% CI)	*p*-Value ^1^
**ASCVD score**	Baseline	17.2% (12.7)	-	-
	End of treatment	17.7% (12.9)	+0.51% (−3.95, 4.97)	0.50
	one year	16.9% (14.1)	−0.3% (−5.3, 4.7)	0.76
	two years	17.1% (12.3)	−0.08% (−5, 4.83)	0.11
	three years	17.2% (15.7)	+0.08% (−7.16, 7.32)	0.68
**FIB-4 score**	Baseline	3.29 (2.68)	-	-
	End of treatment	2.46 (1.61)	−0.83 (−1.48, −0.18)	<0.001 *
	one year	2.6 (1.86)	−0.69 (−1.37, −0.01)	<0.001 *
	two years	2.38 (1.3)	−0.91 (−1.54, −0.29)	<0.001 *
	three years	2.53 (1.83)	−0.76 (−1.48, −0.03)	<0.001 *

ASCVD, Atherosclerotic Cardiovascular Disease 2013; FIB-4, fibrosis-4 index. *: significant p-value (<0.05). ^1^ *p* values represent the mean difference from baseline in ASCVD or FIB-4 scores. Changes from baseline are compared by unadjusted repeated measures linear regression.

**Table 4:** Association of ASCVD and FIB-4 scores over follow-up time points (N = 50).

Coefficients	Estimate	S.E.	Wald	*p*-Value ^1^
**Intercept**	15.396	2.663	33.44	<0.001 *
**Time**				
End of treatment	−1.369	1.063	1.66	0.2
1 year	−1.793	1.317	1.86	0.17
2 years	0.221	1.606	0.02	0.89
3 years	−2.553	1.597	2.56	0.11
**BMI**				
overweight or obese	2.215	2.745	0.65	0.42
**Creatinine**				
>1 mg/dL	7.252	2.501	8.41	0.0037 *
**FIB-4 score**	−0.324	0.361	0.80	0.37
**FIB-4 score: Time**				
FIB-4 score: End of treatment	0.422	0.356	1.40	0.24
FIB-4 score: 1st year	1.161	0.485	5.73	0.0166 *
FIB-4 score: 2nd year	1.073	0.592	3.28	0.07
FIB-4 score: 3rd year	2.520	0.452	31.13	<0.001 *

ASCVD, Atherosclerotic Cardiovascular Disease 2013; BMI, body mass index; FIB-4, fibrosis-4 index; S.E, standard error. *: significant p-value (<0.05). ^1^ *p* values represent the association between the different variables and ASCVD score, measured at the different time points. Comparison done by repeated measures linear regression.

## Data Availability

The data presented in this study are available on request from the corresponding author.

## Supplementary Materials

The following supporting information can be downloaded at: https://www.mdpi.com/article/10.3390/jcm11195781/s1, Table S1: Summary of Selected studies; Table S2: STROBE checklist; Text S1: Sample size calculation; Table S3: Types of utilized DAA therapy; Table S4. Missing values; Table S5: ASCVD and FIB-4 scores among patients with non-decreasing FIB-4 after treatment; Table S6: Mean ASCVD and FIB-4 scores of patients with acute coronary syndrome; Figure S1: ASCVD score change with time divided by FIB-4 fibrosis groups.

## Abbreviations

ASCVD, Atherosclerotic Cardiovascular Disease 2013; ALT, alanine aminotransferase; AST, aspartate aminotransferase; BMI, body mass index; CHC, chronic hepatitis C; CHD, coronary heart disease; CVD, cardiovascular disease; DAA, Direct acting antivirals; DBP, diastolic blood pressure; FIB-4, fibrosis-4 index; GGT, gamma-glutamyl transferase; HCV, hepatitis C virus; IQR, interquartile range; SBP, systolic blood pressure; SVR, sustained virological response; T2DM, type 2 diabetes mellitus; TE, transient elastography; ULN, upper limit of normal.
