# Supplementary material for: Cardiovascular Risk in Patients with Chronic Hepatitis C Treated with Direct Acting Antivirals
Source: J Clin Med. 2022 Sep 29;11(19):5781. doi: 10.3390/jcm11195781 (PMC9572655; doi:10.3390/jcm11195781)
Supplement: Supplementary file 1 [file jcm-11-05781-s001.zip › jcm-1918841-supplementary.pdf]

Supplementary

Table S1. Summary of Selected studies

Reference	Study type and Sample characteristics	HCV treatment	Follow-up time	CV risk assessment	Outcomes
**Iossa et al. (2021)** **[1]**	• Retrospective cohort<br>• N=49	DAA	Baseline, end of treatment, 1 and 2 years after treatment	NI: Tr/HDL, visceral adiposity index, FLI	• No change in CV risk indices<br>• Increased total cholesterol and LDL-C after treatment
**Su et al. (2021)** **[2]**	• Systematic review and meta-analysis<br>• N=309,470, 11 studies	Interferon: 7 studies<br>DAA: 2 studies<br>DAA/ Interferon: 2 studies	-	• CAD<br>• CVD<br>• Stroke	• SVR and antiviral therapy correlated with decreased CVD:<br>• CVD (HR = 0.64)<br>• CAD (HR = 0.73)<br>• stroke (HR = 0.74).
**Butt et al. (2019)** **[3]**	• Retrospective cohort<br>• N= 34,206 (1:1 controlled)	4436 with Interferon, 12,667 with DAA	Up to 10 years	• CAD<br>• PVD<br>• Stroke	• SVR and antiviral therapy associated with decreased CVD<br>• Pegylated interferon and ribavirin (hazard ratio 0.78; 95% CI 0.71–0.85)<br>• DAA regimen (hazard ratio 0.57; 95% CI 0.51–0.65)

**Meissner et al. (2015)** **[4]**	• Post hoc analysis of phase II trial<br>• N=54 with HCV genotype 1	DAA + ribavirin	Weeks 4, 12, 24, 36, and 48	TC, HDL-C, LDL-C, VLDL, triglycerides, and HbA1C	LDL-C concentration increased and triglyceride concentration and VLDL particle size decreased early in therapy
**Carvalho, Velosa, & Serejo (2018)** **[5]**	• Prospective cohort<br>• N=178	73 with Interferon+/-ribavirin, 105 with DAA	One year	lipid, glucose, and iron metabolism	DAA group: ↑TC, ↑LDL-C, ↓TG, stable HOMA Interferon group: ↑TC, ↑ HOMA, ↑TG, stable LDL-C
**Kawagishi et al. (2018)** **[6]**	• Retrospective cohort<br>• N=117	DAA	Week 24 after treatment	Lipid profile	Successful HCV eradication by IFN-free-DAAs decreases CAP and LDL-C in patients with high baseline values
**Graf et al. (2020)** **[7]**	• Prospective cohort<br>• N=46	DAA	Weeks 12, 24 after treatment	Glucose and lipid homeostasis	HCV eradication resulted in a significant increase in TC, LDL-C and HDL-C levels and CAP; HOMA-IR decreased after treatment
**Abdo, Rabiee, Abdellatif, Abdel Alem, & Moustafa (2021)** **[8]**	• Retrospective cohort<br>• N=98 with T2DM	DAA (sofosbuvir and daclatasvir)	Week 12 after treatment	Glucose and lipid homeostasis	Improvement of glycemic state (↓ HOMA-IR) associated with a global worsening of lipid profile (↑TC, ↑LDL-C, ↑HDL-C)
**Huang et al. (2020)** **[9]**	• Prospective cohort<br>• N=617	DAA	Baseline, end of treatment, week 12, 1 and 2 years	Lipid homeostasis and cardio-cerebral diseases	• ↑TC and ↑LDL-C after treatment<br>• Five patients developed cardio-cerebral diseases

			after treatment		during 1376 person-years<br>• LDL-C surge >40% was the only factor predictive of vascular events
**Stine et al. (2017)** **[10]**	• Retrospective cohort<br>• N=175 (26 with available HbA1c values)	DAA	Week 12 after treatment	Glucose homeostasis	• There was no significant difference in pre-treatment to post-treatment HbA1c<br>• 31% of subjects required dose escalation or insulin initiation during treatment.

Table S2. STROBE checklist

	Item No.	Recommendation	Page No.
**Title and abstract**	1	(*a*) Indicate the study's design with a commonly used term in the title or the abstract	4
		(*b*) Provide in the abstract an informative and balanced summary of what was done and what was found	2
**Introduction**			
Background/rationale	2	Explain the scientific background and rationale for the investigation being reported	3
Objectives	3	State specific objectives, including any prespecified hypotheses	3-4
**Methods**			
Study design	4	Present key elements of study design early in the paper	4-5
Setting	5	Describe the setting, locations, and relevant dates, including periods of recruitment, exposure, follow-up, and data collection	4-5
Participants	6	(*a*) *Cohort study*—Give the eligibility criteria, and the sources and methods of selection of participants. Describe methods of follow-up<br>*Case-control study*—Give the eligibility criteria, and the sources and methods of case ascertainment and control selection. Give the rationale for the choice of cases and controls<br>*Cross-sectional study*—Give the eligibility criteria, and the sources and methods of selection of participants	4

		(*b*) *Cohort study*—For matched studies, give matching criteria and number of exposed and unexposed<br>*Case-control study*—For matched studies, give matching criteria and the number of controls per case	NA
Variables	7	Clearly define all outcomes, exposures, predictors, potential confounders, and effect modifiers. Give diagnostic criteria, if applicable	6
Data sources/ measurement	8*	For each variable of interest, give sources of data and details of methods of assessment (measurement). Describe comparability of assessment methods if there is more than one group	*6*
Bias	9	Describe any efforts to address potential sources of bias	6-8
Study size	10	Explain how the study size was arrived at	7
Quantitative variables	11	Explain how quantitative variables were handled in the analyses. If applicable, describe which groupings were chosen and why	7
Statistical methods	12	(*a*) Describe all statistical methods, including those used to control for confounding	7-8
		(*b*) Describe any methods used to examine subgroups and interactions	7-8
		(*c*) Explain how missing data were addressed	7-8
		(*d*) *Cohort study*—If applicable, explain how loss to follow-up was addressed<br>*Case-control study*—If applicable, explain how matching of cases and controls was addressed<br>*Cross-sectional study*—If applicable, describe analytical methods taking account of sampling strategy	7-8
		(*e*) Describe any sensitivity analyses	7-8
**Results**			
Participants	13*	(a) Report numbers of individuals at each stage of study—eg numbers potentially eligible, examined for eligibility, confirmed eligible, included in the study, completing follow-up, and analysed	5,8
		(b) Give reasons for non-participation at each stage	8
		(c) Consider use of a flow diagram	5
Descriptive data	14*	(a) Give characteristics of study participants (eg demographic, clinical, social) and information on exposures and potential confounders	8
		(b) Indicate number of participants with missing data for each variable of interest	8
		(c) *Cohort study*—Summarise follow-up time (eg, average and total amount)	8
Outcome data	15*	*Cohort study*—Report numbers of outcome events or summary measures over time	8-13
		Case-control study—Report numbers in each exposure category, or summary measures of exposure	
		Cross-sectional study—Report numbers of outcome events or summary measures	
Main results	16	(*a*) Give unadjusted estimates and, if applicable, confounder-adjusted estimates and their precision (eg, 95% confidence interval). Make clear which confounders were adjusted for and why they were included	12-14

		(*b*) Report category boundaries when continuous variables were categorized	
		(*c*) If relevant, consider translating estimates of relative risk into absolute risk for a meaningful time period	
Other analyses	17	Report other analyses done—eg analyses of subgroups and interactions, and sensitivity analyses	NA
**Discussion**			
Key results	18	Summarise key results with reference to study objectives	15
Limitations	19	Discuss limitations of the study, taking into account sources of potential bias or imprecision. Discuss both direction and magnitude of any potential bias	17
Interpretation	20	Give a cautious overall interpretation of results considering objectives, limitations, multiplicity of analyses, results from similar studies, and other relevant evidence	16,17
Generalisability	21	Discuss the generalisability (external validity) of the study results	17
**Other information**			
Funding	22	Give the source of funding and the role of the funders for the present study and, if applicable, for the original study on which the present article is based	NA

*Give information separately for cases and controls in case-control studies and, if applicable, for exposed and unexposed groups in cohort and cross-sectional studies.

Paired t test power calculation

n = 47.17
d = 0.4167
sig.level = 0.05
power = 0.8
alternative = two.sided

NOTE: n is number of *pairs*

Sample size: 47*2= 94 subjects.
Including the 15% drop-out assumption: 94/ (1-0.15) =111
Design effect: D = [1 + (k-1) ρ] = 1+(5-1) *0.5=3.

Final sample size: 111*3/5 = 66 per time point.

Text S1. Sample size calculation

Table S3. Types of utilized DAA therapy

**DAA Type**	**n**

**Elbasvir + Grazoprevir**	7
**Ombitasvir/Rit + Paritaprevir + Dasabuvir +/- Ribavirin**	16
**Glecaprevir/Pibrentasvir**	17
**Sofosbuvir + Velpatasvir +/- Ribavirin**	22
**Sofosbuvir + Daclatasvir +/- Ribavirin**	12
**Sofosbuvir + Ledipasvir +/- Ribavirin**	9
**Sofosbuvir + Ribavirin**	5
**Sofosbuvir + Simeprevir +/- Ribavirin**	2
**Sofosbuvir + Velpatasvir + Voxilaprevir**	1
**Total**	91

Table S4. Missing values.

Variable	**Baseline, N=91**	**End of DAA treatment, N=91**	**one year after DAA, N=88**	**two years after DAA, N=81**	**three years after DAA, , N=60**
Systolic blood pressure	1 (1%)	2 (2%)	2 (2%)	5 (6%)	5 (8%)
diastolic blood pressure	1 (1%)	2 (2%)	2 (2%)	5 (6%)	4 (7%)
Total Cholesterol	12 (13 %)	15 (16 %)	16 (18 %)	14 (17 %)	9 (15 %)
LDL-C level (mg/dL)	39 (43 %)	74 (81 %)	50 (57 %)	33 (41 %)	21 (35 %)
HDL-C level (mg/dL)	17 (19 %)	17 (19 %)	19 (22 %)	20 (25 %)	12 (20 %)
ASCVD score	24 (26.37 %)	29 (31.87 %)	38 (43.18 %)	40 (49.38 %)	36 (60 %)

Table S5. ASCVD and FIB-4 scores among patients with non-decreasing FIB-4 after treatment

**Variable**	**Baseline[1] N=42**	**End of DAA treatment[1] N=42**	**one year after DAA[1] N=42**	**two years after DAA[1] N=36**	**three years after DAA[1] N=28**	**p-value[2]**
ASCVD	18 (15)	17 (13)	16 (16)	16 (13)	14 (10)	>0.9
missing	13	17	19	21	20	

FIB4 score	2.4 (2.1)	2.35 (1.4)	2.8 (2.3)	2.2 (0.8)	2.4 (1.43)	0.6

[1]Mean (SD)
[2]Kruskal-Wallis rank sum test

Table S6. Mean ASCVD and FIB-4 scores of patients with acute coronary syndrome

	ASCVD					FIB-4				
	**Baseline**	**End of DAA**	**1-yr**	**2-yrs**	**3-yrs**	**Baseline**	**End of DAA**	**1-yr**	**2-yrs**	**3-yrs**
**Patient 1**	17.9%	-	-	-	-	1.4	1.5	1.4	1.2	0.8
**Patient 2**	6.4%	5.6%	12%	-	-	1.9	2.5	2.2	2.4	2.1

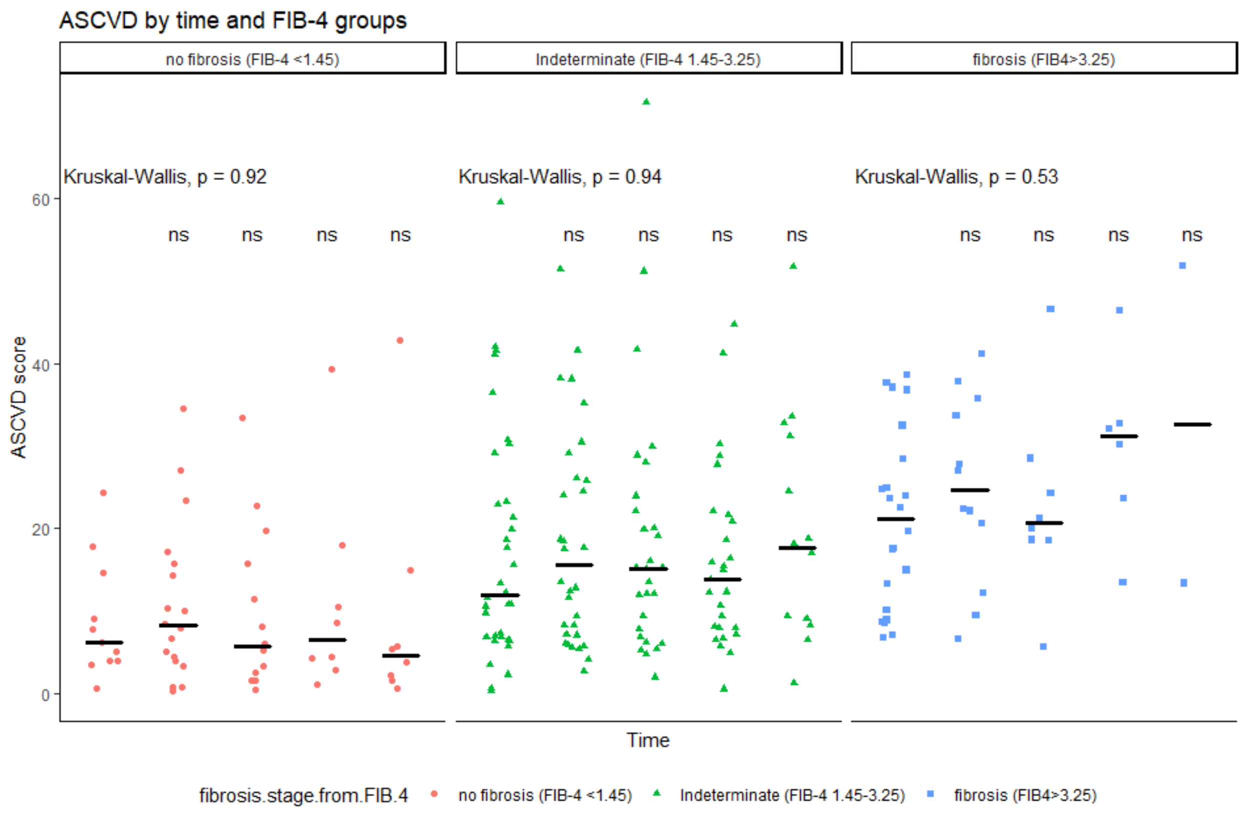

Supplementary Figure S1. ASCVD score change with time divided by FIB-4 fibrosis groups.

1. Iossa, D.; Vitrone, M.; Gagliardi, M.; Falco, E.; Ragone, E.; Zampino, R.; Durante-Mangoni, E. Anthropometric parameters and liver histology influence lipid metabolic changes in HCV chronic hepatitis on direct-acting antiviral treatment. *Ann Transl Med* **2021**, *9*, 35, doi:10.21037/atm-20-669.
2. Su, X.; Zhao, X.; Deng, J.L.; Li, S.N.; Du, X.; Dong, J.Z.; Ma, C.S. Antiviral treatment for hepatitis C is associated with a reduced risk of atherosclerotic cardiovascular outcomes: A systematic review and meta-analysis. *J Viral Hepat* **2021**, *28*, 664-671, doi:10.1111/jvh.13469.
3. Butt, A.A.; Yan, P.; Shuaib, A.; Abou-Samra, A.B.; Shaikh, O.S.; Freiberg, M.S. Direct-Acting Antiviral Therapy for HCV Infection Is Associated With a Reduced Risk of Cardiovascular Disease Events. *Gastroenterology* **2019**, *156*, 987-996.e988, doi:10.1053/j.gastro.2018.11.022.
4. Meissner, E.G.; Lee, Y.J.; Osinusi, A.; Sims, Z.; Qin, J.; Sturdevant, D.; McHutchison, J.; Subramanian, M.; Sampson, M.; Naggie, S.; et al. Effect of sofosbuvir and ribavirin treatment on peripheral and hepatic lipid metabolism in chronic hepatitis C virus, genotype 1-infected patients. *Hepatology* **2015**, *61*, 790-801, doi:10.1002/hep.27424.
5. Carvalho, J.R.; Velosa, J.; Serejo, F. Lipids, glucose and iron metabolic alterations in chronic hepatitis C after viral eradication - comparison of the new direct-acting antiviral agents with the old regimens. *Scand J Gastroenterol* **2018**, *53*, 857-863, doi:10.1080/00365521.2018.1473486.
6. Kawagishi, N.; Suda, G.; Nakamura, A.; Kimura, M.; Maehara, O.; Suzuki, K.; Nakamura, A.; Ohara, M.; Izumi, T.; Umemura, M.; et al. Liver steatosis and dyslipidemia after HCV eradication by direct acting antiviral agents are synergistic risks of atherosclerosis. *PLoS One* **2018**, *13*, e0209615, doi:10.1371/journal.pone.0209615.
7. Graf, C.; Welzel, T.; Bogdanou, D.; Vermehren, J.; Beckel, A.; Bojunga, J.; Friedrich-Rust, M.; Dietz, J.; Kubesch, A.; Mondorf, A.; et al. Hepatitis C Clearance by Direct-Acting Antivirals Impacts Glucose and Lipid Homeostasis. *J Clin Med* **2020**, *9*, doi:10.3390/jcm9092702.
8. Abdo, M.; Rabiee, A.; Abdellatif, Z.; Abdel Alem, S.; Moustafa, A. Impact of sustained virological response on metabolic disorders in diabetic chronic hepatitis C virus patients after treatment with generic sofosbuvir and daclatasvir. *Eur J Gastroenterol Hepatol* **2021**, *33*, 1588-1594, doi:10.1097/meg.0000000000001903.
9. Huang, C.F.; Dai, C.Y.; Yeh, M.L.; Huang, C.I.; Lee, H.C.; Lai, W.T.; Liang, P.C.; Lin, Y.H.; Hsieh, M.Y.; Hou, N.J.; et al. Cure or curd: Modification of lipid profiles and cardio-cerebrovascular events after hepatitis C virus eradication. *Kaohsiung J Med Sci* **2020**, *36*, 920-928, doi:10.1002/kjm2.12275.
10. Stine, J.G.; Wynter, J.A.; Niccum, B.; Kelly, V.; Caldwell, S.H.; Shah, N.L. Effect of Treatment with Direct Acting Antiviral on Glycemic Control in Patients with Diabetes Mellitus and Chronic Hepatitis C. *Ann Hepatol* **2017**, *16*, 215-220, doi:10.5604/16652681.1231581.
